# Modeling to Predict Cases of Hantavirus Pulmonary Syndrome in Chile

**DOI:** 10.1371/journal.pntd.0002779

**Published:** 2014-04-24

**Authors:** Elaine O. Nsoesie, Sumiko R. Mekaru, Naren Ramakrishnan, Madhav V. Marathe, John S. Brownstein

**Affiliations:** 1 Children's Hospital Informatics Program, Boston Children's Hospital, Boston, Massachusetts, United States of America; 2 Department of Pediatrics, Harvard Medical School, Boston, Massachusetts, United States of America; 3 Network Dynamics and Simulation Science Laboratory, Virginia Bioinformatics Institute, Virginia Tech, Blacksburg, Virginia, United States of America; 4 Department of Computer Science, Virginia Tech, Blacksburg, Virginia, United States of America; 5 Department of Epidemiology, Biostatistics and Occupational Health, McGill University, Montreal, Canada; Tulane School of Public Health and Tropical Medicine, United States of America

## Abstract

**Background:**

Hantavirus pulmonary syndrome (HPS) is a life threatening disease transmitted by the rodent *Oligoryzomys longicaudatus* in Chile. Hantavirus outbreaks are typically small and geographically confined. Several studies have estimated risk based on spatial and temporal distribution of cases in relation to climate and environmental variables, but few have considered climatological modeling of HPS incidence for monitoring and forecasting purposes.

**Methodology:**

Monthly counts of confirmed HPS cases were obtained from the Chilean Ministry of Health for 2001–2012. There were an estimated 667 confirmed HPS cases. The data suggested a seasonal trend, which appeared to correlate with changes in climatological variables such as temperature, precipitation, and humidity. We considered several Auto Regressive Integrated Moving Average (ARIMA) time-series models and regression models with ARIMA errors with one or a combination of these climate variables as covariates. We adopted an information-theoretic approach to model ranking and selection. Data from 2001–2009 were used in fitting and data from January 2010 to December 2012 were used for one-step-ahead predictions.

**Results:**

We focused on six models. In a baseline model, future HPS cases were forecasted from previous incidence; the other models included climate variables as covariates. The baseline model had a Corrected Akaike Information Criterion (AICc) of 444.98, and the top ranked model, which included precipitation, had an AICc of 437.62. Although the AICc of the top ranked model only provided a 1.65% improvement to the baseline AICc, the empirical support was 39 times stronger relative to the baseline model.

**Conclusions:**

Instead of choosing a single model, we present a set of candidate models that can be used in modeling and forecasting confirmed HPS cases in Chile. The models can be improved by using data at the regional level and easily extended to other countries with seasonal incidence of HPS.

## Author Summary

Hantavirus pulmonary syndrome (HPS) is a severe disease present in Chile, Argentina and other countries in the Americas. Mortality rates for HPS can be as high as 60% for some outbreaks. Although hantavirus outbreaks tend to be small, the high death rate, unavailability of a vaccine, and occurrence of infections in rural regions where individuals are least likely to have appropriate healthcare make HPS forecasting an important public health issue in Chile and other countries. We present an approach for modeling and forecasting confirmed HPS cases in Chile. Seasonal time series models that predict future cases based on previous cases appear reasonable. However, adding climate variables such as precipitation, which is thought to indirectly influence outbreaks of hantavirus slightly improves the model fit. To further improve the current models to make them more useful for public health preparedness/interventions, data at the regional level with reliable predictions several months into the future are needed.

## Introduction

Reports indicate that illness from hantaviruses existed as early as the 1950s [1], although the virus was only officially recognized in the Americas in 1993 [1]–[3]. An outbreak of hantavirus in the Four Corners region of the United States led to investigation and isolation of the Sin Nombre virus (SNV). Thereafter, several other hantaviruses with the ability to cause severe human disease were discovered in rodents in the Americas [2], with SNV and Andes virus (ANDV) predominating in North and South America respectively [4]. Rodents are a natural reservoir for hantaviruses. Typically, different rodents carry different hantavirus strains and rodent species vary by geography. The deer mouse (*Peromyscus maniculatus*) is the primary reservoir for SNV [2], [5]–[7], while *Oligoryzomys longicaudatus* commonly known as the long-tailed pygmy rice rat is the primary reservoir for the ANDV [3], [4], [8]–[10]. The long-tailed pygmy rice rat is common in rural Argentina and Chile [9], [11].

Transmission of hantaviruses to humans occurs through contact with infected rodent saliva, excreta or inhalation of contaminated aerosol, and, rarely, through the bite of an infected rodent [12]–[14]. Outbreaks are typically small and geographically confined partly due to the lack of human-to-human transmission. Infections can progress to severe disease defined by geographical regions: hemorrhagic fever with renal syndrome (HFRS) in the Old World (Asia and Europe) and hantavirus pulmonary syndrome (or hantavirus cardiopulmonary syndrome) (HPS) in the New World (Americas) [4]. Symptoms of HPS include myositis, fever, breathing difficulties, and in some cases, vomiting, diarrhea, headaches, dizziness and kidney problems [13]–[15]. There are an estimated 300 cases of HPS per year in the Americas [15] and mortality rates for HPS have been as high as 60% for some outbreaks [13], [16]. However, case fatality ratio differs by virus strain.

ANDV, which was first identified in Chile's Cochamo, Los Lagos Region in 1995 is the main cause of HPS in the country and throughout South America [17]. Clinical infections are usually identified in rural regions in the central and southern regions of Chile. Several social and physical factors influence hantavirus transmission. Factors such as temperate forestation, farm settlements, degraded housing, breakdown of sewage systems and inadequate sanitation and waste disposal facilities can contribute to rodent infestation [18]. Increased rodent reservoir populations have been associated with increased human risk of hantavirus infections [16], [19] Variations in rodent populations correlate with seasonality and climate variables (such as precipitation and temperature) as well as landscape alteration (such as that due to farming) [20], [21]. Despite several theories, the exact relationship between climate and rodent abundance remains unknown.

To better understand the population dynamics of relevant rodent colonies, researchers have investigated population spatial distribution and dynamics of transmission agents [20], [22], spatial and temporal distribution of cases in relation to other variables [23], hantavirus risk estimation [24], [25], and climatic aspects of transmission [26]. Models have also illustrated that alternation of seasons could lead to outbreaks of hantavirus [2] and disappearance and reappearance of hantavirus has been observed with changes in environmental conditions [27]. Based on these observations and the paucity of studies from South America that predicts HPS, we explore the influence of climatological variables in models for forecasting HPS cases in Chile. The inclusion of climatological variables slightly improves the model fit and prediction accuracy in some cases. Specifically, the addition of a combination of precipitation and temperature into the time series models appear to improve prediction of the case trend, which reinforces the hypothesis that changes in climatic conditions especially precipitation could have an influence on incidence.

## Materials and Methods

### Data Sources

#### Official case count time series

**Figure 1:**
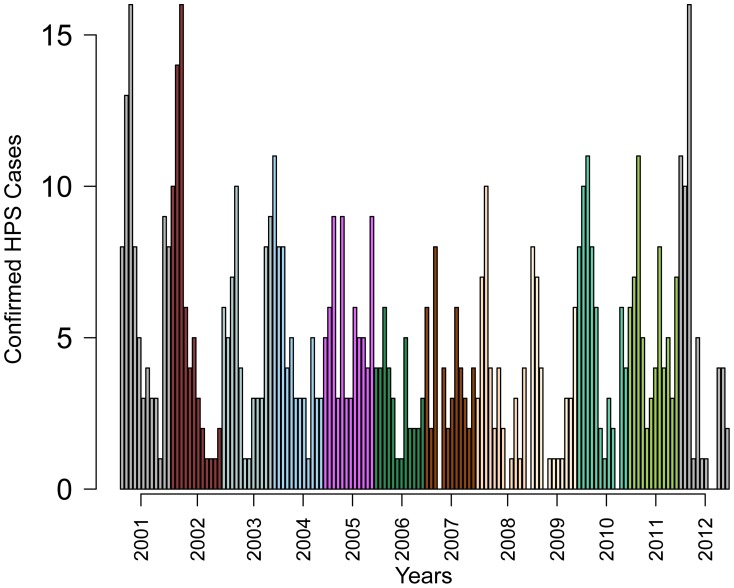
Confirmed HPS cases in Chile for 2001–2012. Different colors indicate different years and each bar represents a month starting from January of 2001.

Publicly available data on confirmed HPS cases were obtained from the Chilean Ministry of Health (http://epi.minsal.cl). We extracted the data from reports presented on the Ministry of Health (MOH) website. The data spanned January 2001 to December 2012 and was available at a monthly resolution (see Figure 1). At the time of this writing, the HPS map on the Chile's MOH site showed a cumulative distribution of cases from 2007 to the week of May 26th, 2012. The case distribution from 2007–2012 by region was as follows: Los Ríos (105), Biobío (92), Maule (59), Los Lagos (47), Del Libertador B. O'Higgins (44), Araucanía (41) and Valparaíso (6) [28]. The number of reported HPS cases for this period were also compared and found to be equatable to the number of reported HPS cases extracted from HealthMap [29] - an automated online system for real-time disease outbreak monitoring and surveillance based on informal sources such as news reports.

#### Climate data

**Figure 2:**
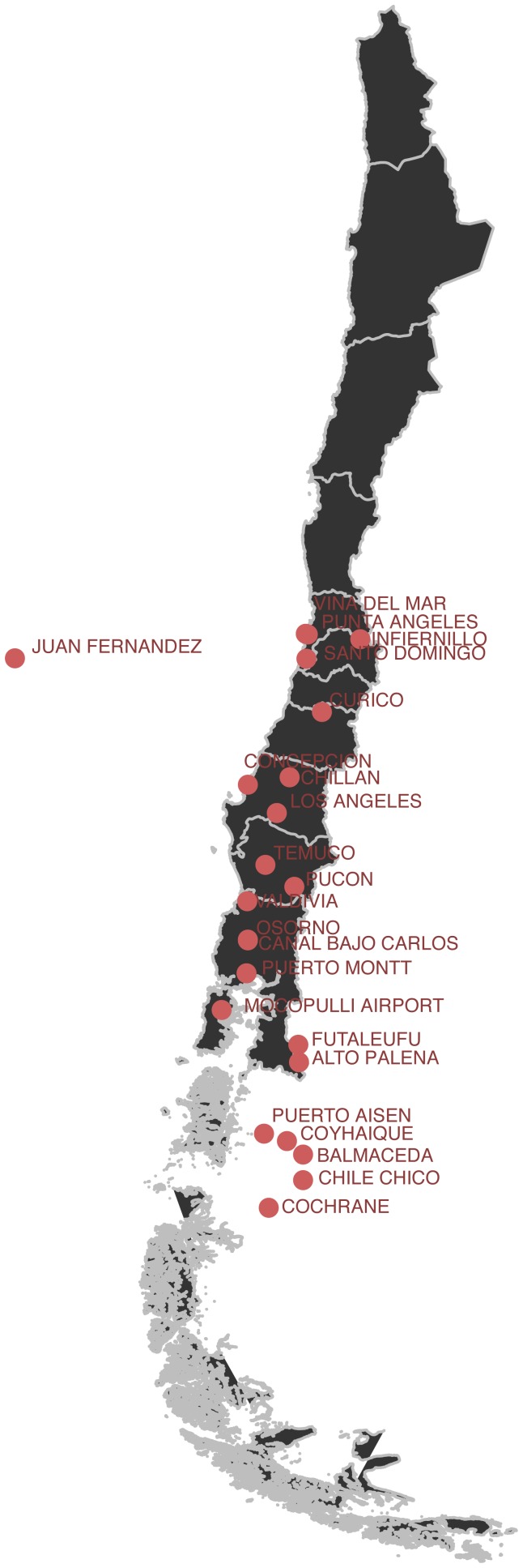
Locations of weather stations that fall in regions with confirmed HPS cases from 2007–2012.

Climate data were obtained from the Global Historical Climatology Network (GHCN) of the US National Oceanic and Atmospheric Administration (NOAA) [30]. GHCN data was available for nine weather stations located across nine of the fifteen regions in Chile. We further restricted the data to stations located in regions with incidence of HPS. The GHCN data contained temperature and cumulative precipitation. Temperature was recorded in degree Celsius, and the average mean, maximum and minimum monthly values were presented. Precipitation was measured as total precipitation per month and presented at tenths of millimeter precision. Data on daily relative humidity and dew point were downloaded from NOAA's National Climatic Data Center at a daily resolution. We chose 23 stations (see Figure 2) located in Los Lagos, Maule, Valparaíso, Biobío, Araucanía, Los Lagos and Aisen. We also downloaded the data from January 2001 to December 2012 to correspond with the time range of the reported HPS cases. In addition, monthly averages were estimated from the reported daily relative humidity and dew point. These climatological variables were chosen based on available data and previous studies on spatial spread of hantaviruses using similar explanatory variables [22], [26].

### Model Fitting

As stated, the aim of this study was to build models that estimate and forecast HPS activity in Chile, in addition to exploring the influence of climatological variables in the models. We selected six time series models after considering several Auto Regressive Integrated Moving Average (ARIMA) and regression with ARIMA error models. ARIMA models are useful for modeling lagged relationships typically present in periodically collected data [31]. Also, a lagged relationship between climate variables and cases has biologic plausibility as these variables may have lagged effect on rodent population and behavior and through that pathway a lagged effect on cases. ARIMA models have been used in modeling and forecasting infectious diseases such as influenza [32] and dengue [33]. We briefly describe these modeling approaches.

#### ARIMA models

ARIMA models belong to the Box-Jenkins approach to time-series modeling [34] and are represented as ARIMA(p,d,q), where p indicates the autoregressive (AR) order, d the differencing order and q the moving average (MA) order. ARIMA models assume a stationary time series, implying that the data vary around a constant mean and variance over time. Nonstationary time series variables can be made stationary by transformation or differencing. An ARIMA(p,d,q) model can be written as:
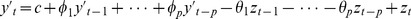
(1)where c is a constant, 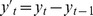 represents the differenced series, 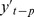
are lagged values and 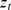 is a white noise process. Model (1) can be written using the backshift operator *L*, which represents lagging of the data by a single time period (e.g. one month). Similarly *L^2^* implies the data is lagged by two time periods or 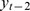. The first difference using the shift operator can be presented as 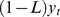 and *d^th^*-order difference can be written as 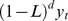. Using this notation, equation (1) can be written as:
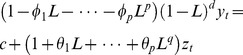
(2)where d represents the number of differences, q are the number of MA terms, p is the number of AR terms and *φ* and θ are coefficients.

We also fit seasonal ARIMA models, defined as ARIMA(p,d,q)(P,D,Q)_s_ where P is the number of seasonal autoregressive terms, D represents the number of seasonal difference, Q is the number of seasonal moving average terms and s is the number of periods in the season which was 12 in this study since HPS cases were reported at a monthly resolution. For example an ARIMA(1,1,1)(1,1,1)_4_ without a constant can be written as:
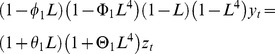
(3)where *(1−φ_1_L)* is the non-seasonal AR(1) term, *(1−*Φ***_1_*** *L^4^)* is the seasonal AR(1) term, *(1−L)* is the non-seasonal difference, *(1−L^4^)* is the seasonal difference term, *(1+θ* ***_1_*** *L)* is the non-seasonal MA(1) term and *(1+*Θ*_1_L^4^)* is the seasonal MA(1) term. More information on ARIMA models can be found in several textbooks (for examples see [34] and [35]).

#### Regression models with ARIMA errors

Regression with ARIMA errors basically combines regression and ARIMA models. In general the model can be written as:
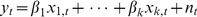
(4)where *n_t_* is assumed to follow an ARIMA model. For instance, if *y_t_* and *x_t_* are differenced, then a regression model with ARIMA(1,1,1) errors can be written as:
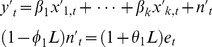
(5)where 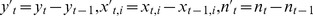 and *e_t_* is a white noise series.

#### Analysis

Initially, we fitted a simple time series regression model to evaluate the potential impact of the climate variables on HPS cases. The residuals of each fitted model were evaluated and if the observed residuals did not correspond to white noise, we employed a three step modeling approach which involved selecting a candidate model; estimating the model and performing diagnostic tests and lastly, forecasting future observations.

Autocorrelation function (ACF) and partial autocorrelation function (PACF) plots were used to determine possible values for the AR and MA orders. AR and MA coefficients were estimated using conditional-sum-of-squares and maximum likelihood. Additionally, we also used the cross-correlation function (CCF) to identify lags of the climatological variables to include in the models. We only used lags up to 4 months due to the length of the time series. CCF was calculated between pre-whitened time series for each of the climate variables and the confirmed HPS cases. The variables were pre-whitened by first fitting an ARIMA model to the time-series of each of the climate variables. Next, the HPS case data were filtered using the estimated coefficients for each of the fitted models. Lastly, we assessed correlations between the residuals from each of the models fitted to the climate variables and the filtered confirmed HPS cases using the CCF. Pre-whitening is necessary when CCF values are affected by the time series structure of the covariates and existing common trends between the covariates and the response series over time [35]. Dew point was excluded from the modeling process because it did not have a significant CCF with the HPS cases. Models consisting of one or more of the remaining climate variables in addition to lagged climate variables were considered.

We adopted an information-theoretic approach for model and ranking. Model selection was based on a minimization of the Corrected Akaike Information Criterion (AICc) [36]–[38] and diagnostic checks. Diagnostic checks involved examining the residuals for each fitted model for autocorrelations and randomness. The difference (ΔAICc_i_) between the AICc_i_ of each selected model and the minimum AICc was also calculated. Furthermore, we estimated model likelihoods and Akaike weights *w_i_* as a measure of the strength of evidence in support of each of the candidate models given the data and the selected models. The weight was calculated as follows: 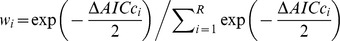 [37]. Here, R represents the number of candidate models. Lastly, we estimated the evidence ratio between the model with minimum AICc and the model without climate variables by taking the ratio of the Akaike weights.

The monthly HPS case data were divided into two sets: one for the fitting process and the other for validation. Data from 2001–2009 were used in fitting. Data from January 2010 to December 2012 were used in validation; 106 data points were used in fitting and 36 for evaluation. The selected models were also used in one-step-ahead predictions of the evaluation points and prediction accuracy was assessed based on the coefficient of variation R^2^ and the Root Mean Squared Error (RMSE). The modeling process was implemented using the TSA and forecast packages in R [39]. A sample code is given in the Supplementary Information S1.

## Results

**Figure 3:**
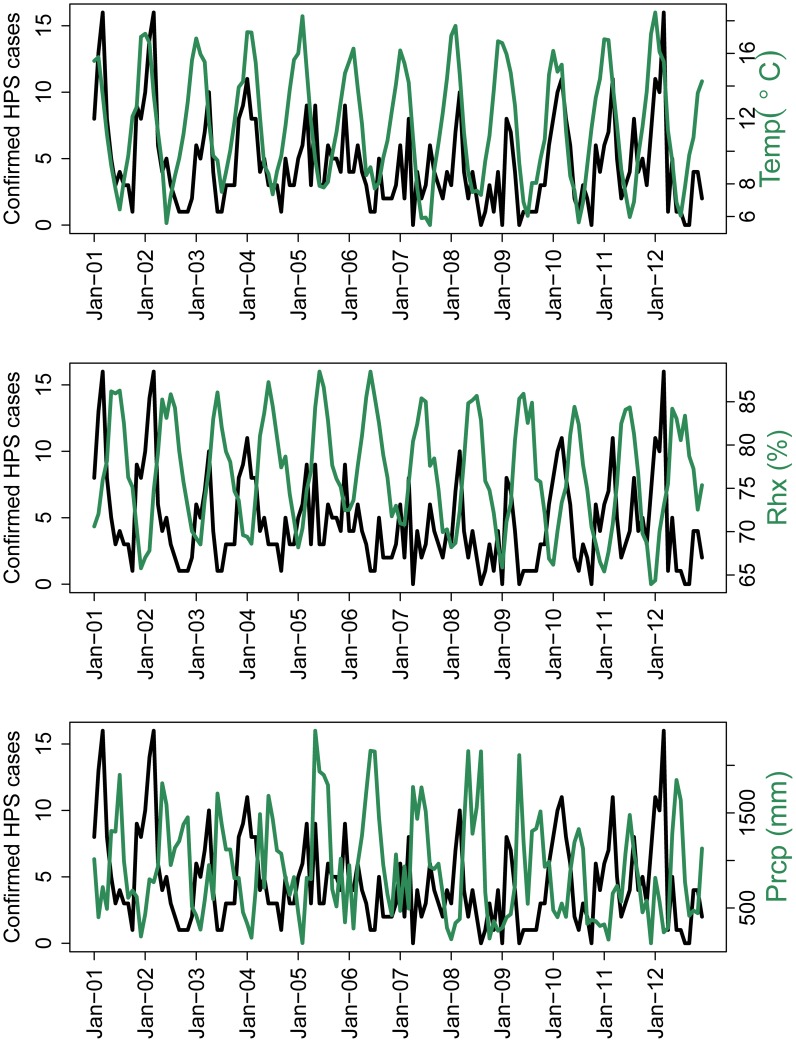
Confirmed HPS cases in Chile and climate variables from 2001–2012. Abbreviations: Temp = Temperature, Rhx = Relative Humidity and Prcp = Precipitation. The data is at a monthly time scale.

From 2001–2012, there were an estimated 667 cases of HPS with an average of 56 cases per year. HPS cases were relatively consistent among years with seasonal peaks between January and April as shown in Figure 1. The epidemic peaks appeared to be preceded by peaks in mean temperature. In addition, increases in precipitation and relative humidity appeared to correlate with troughs in confirmed HPS cases (see Figure 3). Cross-correlations between pre-whitened time series of climate variables and the confirmed HPS case data were significant at different lags, mostly between lags 1 and 4. The cross-correlations of variables used in the modeling process are shown in Table 1.

**Table 1:** Cross-correlations between climate time series and reported HPS cases.

Variable	Lag in months										
	0	1	2	3	4	5	6	7	8	9	10
**Tmin**	−0.158	**−0.199**	−0.022	−0.102	−0.166	−0.151	0.005	−0.066	−0.060	0.004	−0.153
**Tmax**	0.018	−0.039	−0.094	0.063	**−0.187**	−0.025	−0.111	0.035	−0.123	0.118	−0.058
**Prcp**	−0.012	**0.175**	−0.019	−0.008	**0.188**	0.021	0.019	−0.037	0.026	−0.131	−0.161
**Rhx**	−0.070	**−0.311**	0.008	−0.042	**−0.197**	−0.052	0.100	−0.002	**−0.204**	0.047	−0.005

Tmin represents monthly average minimum temperature, Tmax is the monthly average max temperature, Prcp is cumulative monthly precipitation and Rhx is relative humidity. Each variable was fitted to an ARIMA model and the HPS case time series data was filtered using the estimated coefficients from each of the fitted models. Correlations were then assessed between the residuals from each of the models fitted to the climate variables and the filtered confirmed HPS cases using the cross-correlation function. Significant correlations are in **bold**.

Five models were selected in addition to the model based solely on previous HPS cases (baseline model). The models are presented based on AICc rankings in Table 2. The model with precipitation with lags at one and four months was the most parsimonious with a weight of 0.63. The second and third ranked models should also be considered relevant, owing to ΔAICci<4 [38]. All three ‘best’ models included precipitation at one and four month lags suggesting that this variable is an important predictor variable to model and forecast HPS cases.

**Table 2:** The models are ranked based on AICc and presented by rank order.

Model Errors	Fit			Prediction		AR	MA	Environmental Variables	
	AICc	Δ AICc_i_	w_i_	RMSE	R^2^	Est.	Est.	Vars	Est.
ARIMA(1,0,0)(0,1,1)_12_	437.62	0	0.630	2.557	0.559	0.3655	−0.6781	Prcp(lag 1)	−2.00E-04
								Prcp(lag 4)	1.00E-04
ARIMA(2,0,0)(0,1,1)_12_	440.30	2.68	0.165	2.774	0.551	0.2634	−0.7965	Rhx(lag 1)	−0.3269
						0.2754		Prcp(lag 1)	0.0017
								Prcp(lag 4)	0.0011
								Tmin(lag 1)	−0.4973
ARIMA(2,0,1)(0,1,1)_12_	440.90	3.28	0.122	2.542	0.558	0.2866	−0.7843	Prcp(lag 1)	0.0015
						0.3025	0.1791	Prcp(lag 4)	9.00E-04
								Tmin(lag 1)	−0.8292
ARIMA(2,0,0)(0,1,1)_12_	443.31	5.69	0.037	2.421	0.594	0.2698	−0.745	Tmin(lag 1)	−0.4517
						0.2342			
ARIMA(2,0,0)(0,1,1)_12_	443.64	6.02	0.031	2.438	0.591	0.3099	−0.7507	Prcp(lag 1)	9.00E-04
						0.2044			
**ARIMA(1,0,0)(0,1,1)_12_**	**444.98**	**7.36**	**0.016**	**2.560**	**0.555**	**0.3617**	**−0.6928**		

The errors terms for each of the regression with autoregressive integrated moving average errors models is presented as ARIMA(p,d,q)(P,D,Q)_s_ where p indicates the autoregressive (AR) order, d the differencing order, q the moving average (MA) order, and P, D, Q are the seasonal equivalents, and s is the seasonal period. The Akaike ΔAICc_i_ is the difference between each AICc_i_ and the minimum AICc. The weight *w_i_* represents the probability of each model given the data and the other candidate models in the set. Prediction accuracy is evaluated based on the R^2^ and RMSE. In addition, coefficients for each of the terms in the model in addition to the error terms are presented. Abbreviations: Rhx is relative humidity, Tmin is the minimum temperature, and Prcp is precipitation. The baseline model, which is based solely on previous confirmed cases of HPS is **bold** in the table.

**Figure 4:**
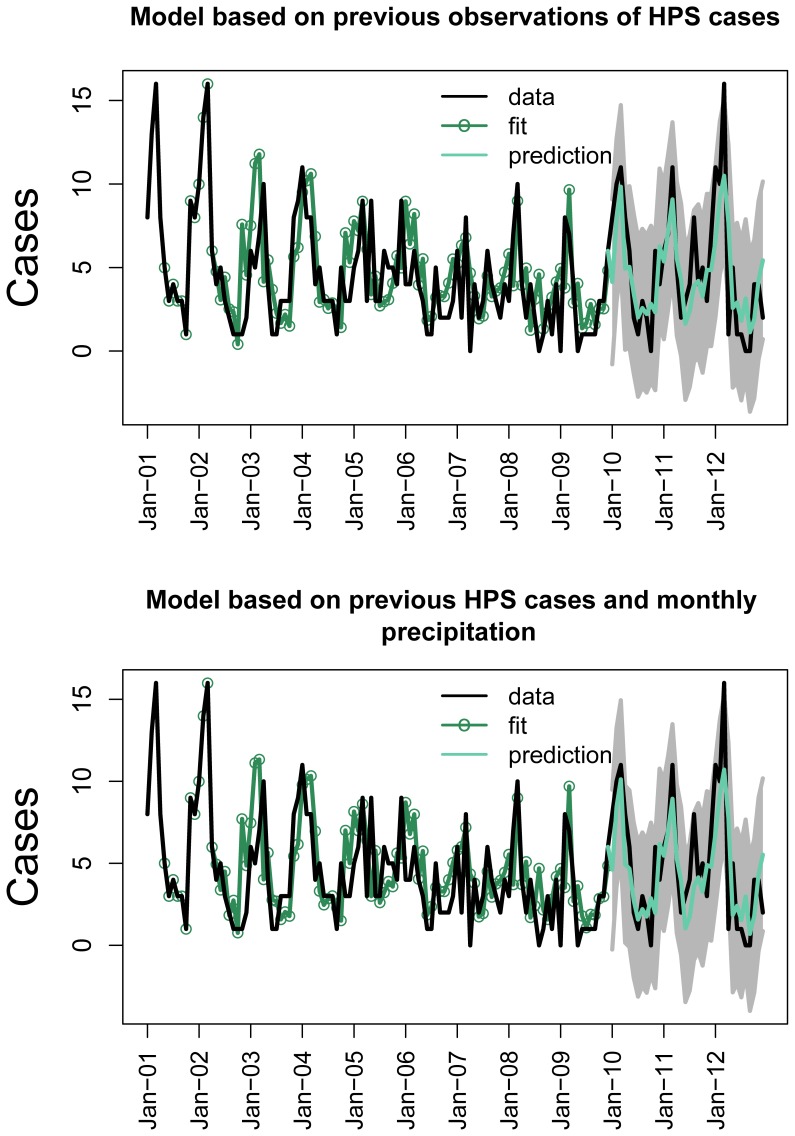
Data fit and forecasting using a model that predicts future cases of HPS based on previous observations and model with the minimum AICc that combines information on previous HPS cases with precipitation data. Data from 2001–2009 was used in fitting, while data from 2010–2012 was used to evaluate the fitted models. There are slight differences in the fit between the two models, but the prediction RMSE is almost identical. The gray area represents the 95% CIs around the predicted values and the data is at a monthly time scale.

Based on the coefficient of variation (R-squared), lagged precipitation in the top ranked model produced a 1.86% improvement in model fit over the baseline model (baseline model R^2^ = 0.484, top ranked model R^2^ = 0.493). We present the fit and forecast of the top ranked model and the baseline model in Figure 4. The predicted number of cases based on both models captured the timing of the peaks observed from 2010–2012 (baseline model R^2^ = 0.555, top ranked model R^2^ = 0.559). Each of the predicted values were captured within the 95% CIs except for the major peak observed in 2012.

## Discussion

In this study, we present several time series models for HPS activity with the inclusion and exclusion of climatological covariates. Univariate models with a seasonal component appear to capture the trend observed in the data and also have a low prediction error suggesting that HPS cases could be predicted one-month ahead. The inclusion of climatological variables slightly improves the model fit and prediction accuracy in some cases. Since HPS cases are typically low, slight deviations in fit and prediction could be significant. The model based on previous observations of HPS cases and precipitation is ranked highest based on the AICc. Increased rainfall may increase the availability of food resources for rodents, which may lead to higher reproduction. Growth in rodent populations may lead to competition for food resources, which may also lead to greater dispersal thereby increasing the likelihood of contact between rodents and humans [21]. As this process takes place overtime, it may partially explain some of the significant correlations observed between lags of the pre-whiten precipitation variable and the confirmed HPS case time series data.

Though some studies have evaluated climate in relation to rodent population dynamics, to our knowledge, there are no published studies on forecasting HPS activity using climate variables for Chile. A study on forecasting HFRS incidence in China used a similar time series modeling approach [31]. However, the authors did not consider the inclusion of climate variables in their model. The availability of climate data in near real-time makes a reasonable addition to the modeling and forecasting of HPS cases.

Climate most likely influences rodent populations based on a combination of weather variables [7], [20], [21], [40]. However, there is a lack of in-depth understanding on how climate change would influence rodent host populations and disease outbreaks. Recent changes in rodent populations and hantavirus infections have been connected with climate change in Europe, though the effects appear to differ from one location to another [3]. However, the natural history and hosts dynamics of hantaviruses suggest that climate change could result in increased incidence in humans, which might also be attributable to changes in human behavior [3], [8], [40]. In addition to the possible elimination of reservoir hosts, increases in the frequency and intensity of extreme climatic events such as droughts and floods, could lead to alteration of hantavirus species composition [8], [41]. A more in-depth understanding of both reservoir rodent population dynamics and disease outbreaks in relation to climate is therefore important for better understanding and modeling of ANDV infections in rodents.

The temporal and spatial resolutions of the available data are limitations of this study. Data at the daily or weekly resolution would be useful. In addition, averaging climate variables across different regions with different levels of HPS cases results in loss of information. Regional modeling and forecasts would be beneficial since weather varies across regions. This loss of information could have affected the contribution of the climate variables to the various models. The models also fail to account for several other factors that could possibly influence rodent populations and human and subsequently hantavirus infections. Other climate variables apart from humidity, precipitation, and temperature might also interact to influence observed cases. In addition, climate data is also available only for some of the regions with high HPS incidence and the locations of weather stations might not be the same as locations with confirmed cases. Furthermore, HPS cases typically occur in rural areas suggesting that not all cases might be reported and mild HPS cases might be undiagnosed. Riquelme et al. [9] suggests using epidemiological questionnaires to improve monitoring and diagnosis of HPS. There is also a likely delay in diagnosis since the average incubation period for hantavirus infections is between 13–17 days [1], [13] possibly meaning some cases could be misclassified by a month. Lastly, the confidence intervals for the presented models include negative values suggesting that a Poisson time series model might be better suited for this data. However, note, that the predicted cases do not fall below zero, indicating that the models presented could be useful for case count predictions.

Although the proposed models have limitations, they provide a framework for monitoring and forecasting HPS cases. HPS is a serious disease with a high fatality ratio. Modeling and forecasting of expected HPS cases could be extremely useful for public health education and control of incidence. The possibility of person-to-person transmission [10], [42] and potential vulnerability of urban communities makes such a modeling approach relevant. In addition, although several environmental management practices and educational campaigns have been launched to limit activities that contribute to the transmission of hantaviruses, HPS continues to be endemic in Chile [12]. Vaccines are currently unavailable, therefore prevention of hantavirus infection involves elimination of possible habitats for rodents, proper garbage disposal practices, and using protective devices (such as gloves, goggles and respirators) when cleaning poorly ventilated structures such as cabins and ensuring proper maintenance in and around household structures. Most hantavirus infections are thought to result from exposure to aerosolized hantavirus. Modeling and forecasting future HPS cases could be useful for the development and implementation of preventive public health measures. Although the models in this study are developed for Chile, methods and analysis can be extended to other countries with HPS.

These models must be updated with the most recent data regularly since changes in various parameters can affect predicted outcomes. Despite the noted limitations and requirements, these models suggest that reliable forecast of HPS cases in Chile is feasible. Solely using past cases to predict future case is possible due to the seasonal nature of the peaks. However, the inclusion of climate variables at an appropriate resolution can significantly improve the prediction of incidence. Although hantavirus outbreaks tend to be small and geographically confined, the high fatality rate, unavailability of a vaccine and occurrence of infections in rural regions where individuals are least likely to have appropriate healthcare makes HPS forecasting an important public health challenge in Chile and several other countries.

## Supporting Information

Supporting Information S1 **Sample R code for fitting models with ARIMA errors.**
(TXT)

## References

[1] Young JC, Hansen GR, Graves TK, Deasy MP, Humphreys JG, et al (2000) The incubation period of hantavirus pulmonary syndrome. Am J Trop Med Hyg 62: 714–717. 11304061 10.4269/ajtmh.2000.62.714

[2] Buceta J, Escudero C, de la Rubia FJ, Lindenberg K (2004) Outbreaks of Hantavirus induced by seasonality. Phys Rev E 69: 021906. 10.1103/PhysRevE.69.021906 14995490

[3] Klempa B (2009) Hantaviruses and climate change. Clin Microbiol Infect 15: 518–523 doi:10.1111/j.1469-0691.2009.02848.x 19604276 10.1111/j.1469-0691.2009.02848.x

[4] Bi Z, Formenty PBH, Roth CE (2008) Hantavirus Infection: a review and global update. J Infect Dev Ctries 2: 3–23 Available: http://www.jidc.org/index.php/journal/article/view/19736383. 19736383 10.3855/jidc.317

[5] Douglass RJ, Wilson T, Semmens WJ, Zanto SN, Bond CW, et al (2001) Longitudinal studies of Sin Nombre virus in deer mouse-dominated ecosystems of Montana. Am J Trop Med Hyg 65: 33–41. 11504405 10.4269/ajtmh.2001.65.33

[6] Carver S, Trueax JT, Douglass R, Kuenzi A (2011) Delayed density-dependent prevalence of Sin Nombre virus infection in deer mice (Peromyscus maniculatus) in central and western Montana. J Wildl Dis 47: 56–63 doi:10.7589/0090-3558-47.1.56 21269997 10.7589/0090-3558-47.1.56 PMC3073316

[7] Carver S, Kilpatrick AM, Kuenzi A, Douglass R, Ostfeld RS, et al (2010) Environmental monitoring to enhance comprehension and control of infectious diseases. J Environ Monit JEM 12: 2048–2055 doi:10.1039/c0em00046a 20957286 10.1039/c0em00046a PMC2990948

[8] Dearing DM, Dizney L (2010) Ecology of hantavirus in a changing world. Ann N Y Acad Sci 1195: 99–112 doi:10.1111/j.1749-6632.2010.05452.x 20536819 10.1111/j.1749-6632.2010.05452.x

[9] Riquelme R, Riquelme M, Torres A, Rioseco ML, Vergara JA, et al (2003) Hantavirus Pulmonary Syndrome, Southern Chile. Emerg Infect Dis 9: 1438–1443 doi:10.3201/eid0911.020798 14718088 10.3201/eid0911.020798

[10] Padula PJ, Edelstein A, Miguel SDL, López NM, Rossi CM, et al (1998) Hantavirus Pulmonary Syndrome Outbreak in Argentina: Molecular Evidence for Person-to-Person Transmission of Andes Virus. Virology 241: 323–330 doi:10.1006/viro.1997.8976 9499807 10.1006/viro.1997.8976

[11] Levis S, Morzunov SP, Rowe JE, Enria D, Pini N, et al (1998) Genetic Diversity and Epidemiology of Hantaviruses in Argentina. J Infect Dis 177: 529–538 doi:10.1086/514221 9498428 10.1086/514221

[12] Purcell L (2006) Hantavirus in rural Chile. J Rural Trop Public Health 5: 79–87.

[13] Jonsson CB, Figueiredo LTM, Vapalahti O (2010) A Global Perspective on Hantavirus Ecology, Epidemiology, and Disease. Clin Microbiol Rev 23: 412–441 doi:10.1128/CMR.00062-09 20375360 10.1128/CMR.00062-09 PMC2863364

[14] Centers for Disease Control and Prevention: Hantavirus pulmonary syndrome (HPS) (2013). CDC. Available: http://www.cdc.gov/hantavirus/hps/. Accessed 18 February 2013.

[15] Muranyi W, Bahr U, Zeier M, van der Woude FJ (2005) Hantavirus Infection. J Am Soc Nephrol 16: 3669–3679 doi:10.1681/ASN.2005050561 16267154 10.1681/ASN.2005050561

[16] Cao L, Cova TJ, Dennison PE, Dearing MD (2011) Using MODIS satellite imagery to predict hantavirus risk. Glob Ecol Biogeogr 20: 620–629 doi:10.1111/j.1466-8238.2010.00630.x

[17] Toro J, Vega JD, Khan AS, Mills JN, Padula P, et al (1998) An outbreak of hantavirus pulmonary syndrome Chile, 1997. Emerg Infect Dis 4: 687–694. 9866751 10.3201/eid0404.980425 PMC2640255

[18] Frey MT, Vial PC, Castillo CH, Godoy PM, Hjelle B, et al (2003) Hantavirus prevalence in the IX Region of Chile. Emerg Infect Dis 9: 827–832 Available: http://wwwnc.cdc.gov/eid/article/9/7/02-0587.htm. 12890323 10.3201/eid0907.020587 PMC3023426

[19] Yates TL, Mills JN, Parmenter CA, Ksiazek TG, Parmenter RR, et al (2002) The ecology and evolutionary history of an emergent disease: hantavirus pulmonary syndrome. Bioscience 52: 989–998.

[20] Murúa R, González LA, Lima M (2003) Population dynamics of rice rats (a Hantavirus reservoir) in southern Chile: feedback structure and non-linear effects of climatic oscillations. Oikos 102: 137–145 doi:10.1034/j.1600-0706.2003.12226.x

[21] Luis AD, Douglass RJ, Mills JN, Bjørnstad ON (2010) The effect of seasonality, density and climate on the population dynamics of Montana deer mice, important reservoir hosts for Sin Nombre hantavirus. J Anim Ecol 79: 462–470 doi:10.1111/j.1365-2656.2009.01646.x 20015212 10.1111/j.1365-2656.2009.01646.x

[22] Carbajo AE, Pardiñas UFJ (2007) Spatial Distribution Model of a Hantavirus Reservoir, the Long-Tailed Colilargo (Oligoryzomys longicaudatus), in Argentina. J Mammal 88: 1555–1568.

[23] Busch M, Cavia R, Carbajo AE, Bellomo C, Gonzalez Capria S, et al (2004) Spatial and temporal analysis of the distribution of hantavirus pulmonary syndrome in Buenos Aires Province, and its relation to rodent distribution, agricultural and demographic variables. Trop Med Int Health 9: 508–519 doi:10.1111/j.1365-3156.2004.01218.x 15078270 10.1111/j.1365-3156.2004.01218.x

[24] Glass GE, Cheek JE, Patz JA, Shields TM, Doyle TJ, et al (2000) Using Remotely Sensed Data To Identify Areas at Risk For Hantavirus Pulmonary Syndrome. Emerg Infect Dis 6: 238–247. 10827113 10.3201/eid0603.000303 PMC2640870

[25] Glass GE, Shields T, Cai B, Yates TL, Parmenter R (2007) Persistently Highest Risk Areas for Hantavirus Pulmonary Syndrome: Potential Sites for Refugia. Ecol Appl 17: 129–139. 17479840 10.1890/1051-0761(2007)017[0129:phrafh]2.0.co;2

[26] Donalisio MR, Peterson AT (2011) Environmental factors affecting transmission risk for hantaviruses in forested portions of southern Brazil. Acta Trop 119: 125–130 doi:10.1016/j.actatropica.2011.04.019 21605537 10.1016/j.actatropica.2011.04.019

[27] Abramson G (2008) Progress in Mathematical Biology Research. In: Kelly JT, editor. Nova Science Publishers. pp. 219–245.

[28] Ministerio de Salud. Departamento de epidemiologia. (n.d.). Available: http://epi.minsal.cl/epi/html/AtlasInteractivos/Nacionales/Atlas_Hanta_Especial/atlas.html. Accessed 18 February 2013.

[29] Freifeld CC, Mandl KD, Reis BY, Brownstein JS (2008) HealthMap: Global Infectious Disease Monitoring through Automated Classification and Visualization of Internet Media Reports. J Am Med Inform Assoc 15: 150–157 doi:10.1197/jamia.M2544 18096908 10.1197/jamia.M2544 PMC2274789

[30] NOAA National Climatic Data Center (n.d.). Available: http://www.ncdc.noaa.gov/cdo-web/.

[31] Liu Q, Liu X, Jiang B, Yang W (2011) Forecasting incidence of hemorrhagic fever with renal syndrome in China using ARIMA model. BMC Infect Dis 11: 218 doi:10.1186/1471-2334-11-218 21838933 10.1186/1471-2334-11-218 PMC3169483

[32] Soebiyanto RP, Adimi F, Kiang RK (2010) Modeling and Predicting Seasonal Influenza Transmission in Warm Regions Using Climatological Parameters. PLoS ONE 5: e9450 doi:10.1371/journal.pone.0009450 20209164 10.1371/journal.pone.0009450 PMC2830480

[33] Descloux E, Mangeas M, Menkes CE, Lengaigne M, Leroy A, et al (2012) Climate-Based Models for Understanding and Forecasting Dengue Epidemics. PLoS Negl Trop Dis 6: e1470 doi:10.1371/journal.pntd.0001470 22348154 10.1371/journal.pntd.0001470 PMC3279338

[34] Box GEP, Jenkins GM (1970) Time Series Analysis: Forecasting & Control. San Francisco: Holden-Day.

[35] Brockwell P, Davis R (2002) Introduction to Time Series and Forecasting. Springer.

[36] Anderson DR (2008) Model Based Inference in the Life Sciences: A Primer on Evidence. New York: Springer.

[37] Burnham K, Anderson D, Huyvaert K (2011) AIC model selection and multimodel inference in behavioral ecology: some background, observations, and comparisons. Behav Ecol Sociobiol 65: 23–35 doi:10.1007/s00265-010-1029-6

[38] Burnham KP, Anderson DR (1998) Model Selection and Inference: A Practical Information Theoretic Approach. New York: Springer.

[39] R Development Core Team (2009) R: A Language and Environment for Statistical Computing. Vienna, Austria: R Foundation for Statistical Computing. Available: http://www.R-project.org.

[40] Mills JN, Gage KL, Khan AS (2010) Potential influence of climate change on vector-borne and zoonotic diseases: a review and proposed research plan. Environ Health Perspect 118: 1507–1514 doi:10.1289/ehp.0901389 20576580 10.1289/ehp.0901389 PMC2974686

[41] Thibault KM, Brown JH (2008) Impact of an extreme climatic event on community assembly. Proc Natl Acad Sci 105: 3410–3415 doi:10.1073/pnas.0712282105 18303115 10.1073/pnas.0712282105 PMC2265133

[42] Wells RM, Estani SS, Yadon ZE, Enria D, Padula P, et al (1997) An unusual hantavirus outbreak in southern Argentina: person-to-person transmission? Emerg Infect Dis 3: 171–174. 9204298 10.3201/eid0302.970210 PMC2627608

